# A systematic review of publications assessing reliability and validity of the Behavioral Risk Factor Surveillance System (BRFSS), 2004–2011

**DOI:** 10.1186/1471-2288-13-49

**Published:** 2013-03-24

**Authors:** Carol Pierannunzi, Shaohua Sean Hu, Lina Balluz

**Affiliations:** 1 Division of Behavioral Surveillance, Centers for Disease Control and Prevention, 1600 Clifton Road NE, Atlanta, GA 300333, USA; 2 Office of Smoking and Health, Centers for Disease Control and Prevention, 1600 Clifton Road NE, Atlanta, GA 300333, USA; 3 National Center for Environmental Health, Centers for Disease Control and Prevention, 1600 Clifton Road NE, Atlanta, GA 300333, USA

## Abstract

**Background:**

In recent years response rates on telephone surveys have been declining. Rates for the behavioral risk factor surveillance system (BRFSS) have also declined, prompting the use of new methods of weighting and the inclusion of cell phone sampling frames. A number of scholars and researchers have conducted studies of the reliability and validity of the BRFSS estimates in the context of these changes. As the BRFSS makes changes in its methods of sampling and weighting, a review of reliability and validity studies of the BRFSS is needed.

**Methods:**

In order to assess the reliability and validity of prevalence estimates taken from the BRFSS, scholarship published from 2004–2011 dealing with tests of reliability and validity of BRFSS measures was compiled and presented by topics of health risk behavior. Assessments of the quality of each publication were undertaken using a categorical rubric. Higher rankings were achieved by authors who conducted reliability tests using repeated test/retest measures, or who conducted tests using multiple samples. A similar rubric was used to rank validity assessments. Validity tests which compared the BRFSS to physical measures were ranked higher than those comparing the BRFSS to other self-reported data. Literature which undertook more sophisticated statistical comparisons was also ranked higher.

**Results:**

Overall findings indicated that BRFSS prevalence rates were comparable to other national surveys which rely on self-reports, although specific differences are noted for some categories of response. BRFSS prevalence rates were less similar to surveys which utilize physical measures in addition to self-reported data. There is very little research on reliability and validity for some health topics, but a great deal of information supporting the validity of the BRFSS data for others.

**Conclusions:**

Limitations of the examination of the BRFSS were due to question differences among surveys used as comparisons, as well as mode of data collection differences. As the BRFSS moves to incorporating cell phone data and changing weighting methods, a review of reliability and validity research indicated that past BRFSS landline only data were reliable and valid as measured against other surveys. New analyses and comparisons of BRFSS data which include the new methodologies and cell phone data will be needed to ascertain the impact of these changes on estimates in the future.

## Background

Health officials recognize the need for accurate data for purposes of program planning, policy evaluation and estimation of health risk prevalence [1]. Telephone surveys have been a staple of data collection methods, in part due to their efficacy and reduced costs. The behavioral risk factor surveillance system (BRFSS) is a state-based telephone survey coordinated by the Centers for Disease Control and Prevention (CDC). Self-reported information regarding chronic conditions and health risk behaviors is collected throughout the year using telephone survey methods in all 50 states, Washington DC, Guam, Puerto Rico and the US Virgin Islands. More than 400,000 adults complete the survey annually, making the BRFSS the largest telephone survey in the world [2].

Individual states use data from the BFRSS to assess need and plan public health priorities. These data have been essential to states and local jurisdictions and have historically been shown to be useful as sources of information [3]. For many states the BRFSS is the sole source of health and health risk behavior data available to policy makers.

Given the importance of the BRFSS data to its constituent jurisdictions, continuous validation of findings is requisite. CDC, perforce, conducts numerous internal checks on BRFSS data. Independent practitioners have also tested BRFSS reliability and validity within their areas of interest. A comprehensive reliability/ validity study of BRFSS was conducted earlier by Nelson [4] examining articles appearing in peer-reviewed journals through the 1990s. They found that most measures taken from the BRFSS were moderately reliable and valid and that many were highly reliable and valid. Using the Nelson study as a framework to examine reliability and validity studies by topic, this research compiled information on reliability and validity testing of BRFSS data from 2004 through 2011 by a number of researchers in peer- reviewed journals and assesses BRFSS data by question topic.

In its current form, the BRFSS not only produces a large data set covering a number of health risk behaviors, but also provides a number of services to states which are engaged in the process of data collection [5]. Generation of samples, weighting to account for demographic and geographic variables and programming to support report writing are provided to state coordinators and their staffs. Traditionally the BRFSS was based exclusively on landline random digit dialing (RDD) samples of households. Random selection among adults within households was also conducted. In 2008 in response to the growing percentage of cell phone only households in the US [6], cell phone samples were piloted and in 2009 all states included cell phone samples in their data collection process. In 2011 the public release of the BRFSS included both landline and cell phone data for the first time. A second important change in 2011 was the move to a new weighting system which incorporates cell phone data as well as including new variables (education, marital status and home ownership) as controls.

The BRFSS is one of several surveys which compile health data in a variety of modes and methods. Many researchers review BRFSS prevalence indicators in terms of prevalence rates from other surveys which can be used to produce national estimates. These include:

### National health interview study (NHIS)

The NHIS is conducted continuously throughout the year using face-to-face interviews in respondents’ homes. Basic health information is collected for all family members, by proxy if necessary. Additional health and socio-demographic information, including health risk behaviors, is collected, by self-report, from one adult family member [2].

### National health and nutrition examination survey (NHANES)

The NHANES collects information on adults and children and combines face-to-face interviews with physical examination measures. The NHANES has been conducted periodically since the early 1960s. In 1999 the NHANES became a continuous survey with data released every two years [7].

### National survey on drug use and health (NSDUH)

The NSDUH is annually compiled from face-to-face interviews. It focuses primarily on substance abuse among respondents 12 years of age and older [1].

### Current population survey (CPS)

The CPS is conducted by the Bureau of Labor Statistics and the Census Bureau [8]. Data are combined from telephone survey and other modes of collection. Data are published for respondents over 15 years of age.

### National survey of family growth (NSFG)

The NSFG gathers information using personal interviews [9]. Topics include family life, marriage and divorce, pregnancy, infertility, use of contraception, and men's and women's health. Adults and teenagers 15 and over are selected as participants.

Despite studies which support findings from self-reported information [3,10], for some scholars and practitioners self-reported data are perceived to be unreliable estimates of health factor prevalence. Moreover in recent years, telephone survey response rates have declined [5,11]. BRFSS response rates have also declined from medians in the 70–75 percent in the 1980s to a median of 57 percent in 2010 [2], resulting in targeted efforts to improve coverage and reach nonrespondents through the use of new contact methods including cell phones [12,13] and reduction of non-response bias through the introduction of new weighting techniques [14]. Despite these ameliorative steps, it is necessary to review reliability and validity in the BRFSS prior to the 2011 changes in protocols and inclusion of cell phone data.

## Methods

No research effort can result in a comprehensive disclosure of all relevant publications, especially on a publically available dataset which encompasses a wide range of topics. The articles presented here were obtained through an extensive search of publications indices (PubMed, ProQuest, and ScienceDirect). Within each search inquiry, keywords included “BRFSS,” “validity,” and/or “reliability.” Any article which included testing of BRFSS reliability and/or validity was included. Articles which expressed only opinions, without any comparisons or statistical testing were not considered. Given that the purpose of this research was to validate self-reported estimates in an era of declining landline telephone coverage, only those articles which have been published from 2004–2011 were included. Articles were then categorized and are presented in the following topic areas:

1. Access to health care/ general health

2. Immunization, preventive screening, and testing

3. Physical activity measures

4. Chronic disease

5. Mental health measures

6. Overweight and obesity measures

7. Tobacco and alcohol use measures

8. Responsible sexual behavior measures

9. Injury risk and violence

Quality of individual studies may vary significantly. Therefore a scoring rubric was devised to estimate the rigor of the tests of reliability and/or validity found in the literature. Higher rankings on the reliability rubric were achieved by authors who conducted reliability tests using repeated test/retest measures, used multiple samples/populations or multiple time periods. The rubric was also scored higher if authors conducted statistical tests, rather than simply comparing prevalence estimates. Authors who simply tested reliability by noting that results within the BRFSS were internally consistent were ranked lower on the reliability rubric. A similar rubric was used to rank validity assessments. Validity tests comparing the BRFSS to physical measures were ranked highest. Comparing BRFSS validity over time or comparing BRFSS against other self-reported data were ranked lower. Higher ranked assessments of validity and reliability were also characterized by more rigorous statistical comparisons, including the use of sensitivity and specificity measures [15], kappa and other statistics [16] or other statistical comparisons [17]. The rubric provided overall categorical rankings and is not intended to be interpreted as an interval measure of quality estimates. For each of the topics the following information is presented:

1. The number of articles relating to reliability of the BRFSS

2. The number of articles relating to validity of the BRFSS

3. The quality of reliability tests used by authors

4. The quality of validity tests

5. An overall assessment of the literature on reliability and validity of the BRFSS

**Figure 1:**
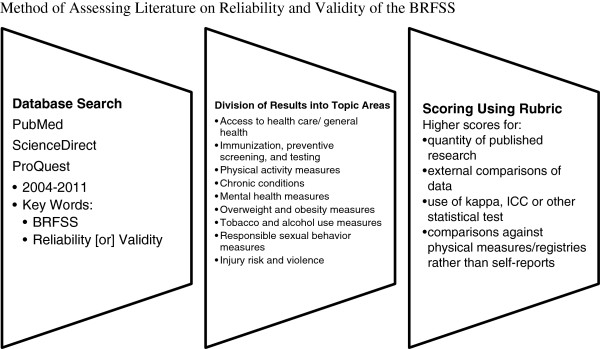
Method of assessing literature on reliability and validity of the BRFSS.

Thus the method used to assess the literature followed the path illustrated in Figure 1.

## Results

The literature provided thirty-two examples of reliability and validity tests published since 2004 for the BRFSS among the ten categories. The literature did not evenly examine each of the topics covered by the BRFSS, and published reports of validity and reliability varied in quality. The largest number of articles was identified for physical activity, access to health care, immunization and preventive testing and diagnoses of chronic disease/conditions (Table 1). Reliability of self-reported diagnoses of chronic conditions between BRFSS and other surveys was found to be high. Virtually all of the test/retest research using BRFSS questions shows high levels of reliability. Reliability for some questions deteriorates when there are longer time periods between administrations of the same questionnaire. For example, respondents were more likely to given the same response when the test/retest administrations were weeks, rather than months, apart [18]. In some cases, self-reports from the BRFSS were highly reliable but prevalence rates differed from other surveys. Physical measure comparisons of self-reported data found that validity of some measures were compromised, especially when reporting on measures of height, weight and health risk associated with sexual behaviors.

**Table 1:** Overall number and ranking of reliability and validity tests for BRFSS estimates

**Topic**	**Number of reliability/ validity tests identified**	**Quality of reliability tests**	**Quality of validity tests**	**Overall assessment of BRFSS validity and reliability**
Access to Health Care/ General Health	4	High (test/retest)	High (comparisons with other surveys and HMO records)	High levels of reliability and validity using published information and test retest methods; BRFSS noted to have higher proportions of persons reporting poorer health.
Immunization and Preventive testing	4	High (test/retest)	High (comparisons with other surveys, national registry data, medical records)	BRFSS rates highly reliable; Validity testing against medical records of individuals high; Validity testing indicating over reporting for some screening tests
Physical activity	8	High (test/retest; time trend)	High (comparisons with other surveys, respondent logs, accelerometers, physical measures)	Better reliability assessment among physically active groups; self-reports substantially reliable; Validity when compared to physical measures moderate
Chronic conditions	4	High (test/retest; comparisons with telephone and written responses)	High (comparisons with physical measures, prescription drug use data, medical history)	High levels of agreement in reliability testing; Some differences in prevalence rates among national surveys when compared to physical measures.
Mental Health Measures	2	High (test/retest with multiple indicators)	N/A	Substantial agreement between test/retest measures
Obesity	3	N/A	Moderate (comparisons against other national surveys, physical measures)	Self-reports reliable across modes; Differences between self-reports and physical measures
Tobacco use	2	N/A	Moderate (comparisons with other national surveys, physical measures)	Self-reports reliable across modes; Differences between self-reports and physical measures
Alcohol/Substance abuse	2	Moderate	Moderate (comparisons with other national surveys)	Trends and risk factors in BRFSS validated by other national surveys; BRFSS prevalence rates lower than other measures at national level and some state levels
Health risk and Sexual behavior	2	N/A	Moderate (comparisons with other national surveys)	BRFSS produces slightly higher level estimates of measures than other national survey; Differences in prevalence rates of HIV testing question.
Injury and violence	1	N/A	Moderate (comparisons with other survey in nonrandom setting)	High levels of agreement between two surveys using nonrandom samples

Validity of BRFSS and other self-reported data was best when respondents were asked about behaviors which were not sensitive, and questions referred to discreet events such as enrollment in health care plans, immunization or testing. In some cases, researchers found BRFSS to be reliable and valid for some groups of individuals and not for others. For example, respondents who reported strenuous physical exercise were found to provide more reliable and valid information than respondents who reported moderate, light or no physical exercise [19]. The categorical rubric produced higher rankings for access to health care, immunization and preventive testing, physical activity and questions on chronic conditions than for other sections of the BRFSS. Results showed similarities between BRFSS and other surveys which utilize self-reported data. These similarities persisted even if self-reports were collected through the use of different modes, such as personal logs, face-to-face interviews and/or telephone interviews. Differences between BRFSS and other surveys was less characterized by mode of self-report than by differences between self-reports and physical measures, as taken by NHANES. More detailed summaries of the articles identified from the literature are presented by topic discussed subsequently.

### Access to health care and general health measures

Several scholars investigated whether self-reported claims of health care coverage can be substantiated (Table 2). Mucci [20] conducted a study asking BRFSS respondents who indicated that they had health insurance to retrieve their insurance cards and validate information provided during the course of the interview. Findings for respondents enrolled in health maintenance organizations (HMOs) showed very high levels of agreement (93% sensitivity). Other respondents were also provided reliable self-reports on health coverage (76% specificity). Respondents were more accurate when reporting that they were enrolled (93%) than reporting correctly on the type of plans (76%) in which they were enrolled. The authors also found higher levels of reliability of health care plan self-reports when respondents had been enrolled for longer periods of time. Clements [21] collected information regarding members in HMO plans from BRFSS respondents as well as collecting respondents’ HMO plan names. Respondents were asked whether they belonged to an HMO. This information was compared with lists of commercial HMOs, by using HMO plan names as reported by respondents. Self-reported membership in HMOs taken from the BRFSS was found to be to be a reliable measure (k = 0.87). These authors also conducted test/retest studies on this question with 78% of respondents reporting the same health care plan information during follow-up interviews.

**Table 2:** Reliability and validity studies of general health assessment and health care access estimates

**First author (year)**	**Focus of research**	**Research findings (confidence intervals)**
Mucci (2006)	Reliability of self-reported health insurance coverage	Self-reports of enrollment agreement .93
		Type of plan self-report reliability .79
Clements (2006)	Reliability of self-reported HMO health care plan	Comparison of self-reports to external data
		***k*** =.87
		Test/retest agreement 78%
Fahimi (2008)	Validity of prevalence of health insurance coverage	No health insurance
		BRFSS (18.4-19.1)*
		NHIS (18.7-20.0)*
		No medical care due to cost
		BRFSS (14.8-15.4)*
		NHIS (7.4-8.2)*
Salomon (2009)	Comparison of trends of general health in BRFSS and other national surveys; Comparison of prevalence of BRFSS and other national surveys	Prevalence of “fair” or “poor” health/ males:
		NHIS (11.3-12.7)*
		BRFSS (15.9-16.8)*
		Prevalence of “fair” or “poor” health/ females:
		NHIS (12.9-14.1)*
		BRFSS (16.6-17.2)*
		BRFSS more likely to show increased proportions of self-reports of “fair” or “poor” health

Fahimi [13] compared confidence intervals for prevalence estimates of no health insurance coverage from BRFSS (18.4 to 19.1) and NHIS (18.7 to 20.0). There were no significant differences in the estimates produced by the two surveys, both of which rely on self-report, albeit using different modes of data collection. Differences were found between BRFSS and NHIS on whether respondents had not sought medical care due to costs (confidence intervals of 14.9 to 15.4 and 7.4 to 8.2, respectively). The BRFSS asked respondents to categorize their general health as “excellent,” “very good,” “good,” “fair” or “poor.” Salomon [22] found that BRFSS and NHIS showed significant differences in the confidence intervals for prevalence of reported “fair” or “poor” self-assessments of health. Time trends of self-reported overall health were also compared in four national surveys: the BRFSS, NHANES, CPS and NHIS. BRFSS respondents reported poorer overall health than other surveys. These scholars suggest that these differences may be attributable to under coverage of cell phone populations, which tend to be younger. However all surveys reviewed by these authors, including the BRFSS, consistently showed that the proportion of respondents indicating that their general health is “excellent” was declining.

### Immunization and prevention/ screening measures

Several measures of immunization, preventive screening and testing are collected by the BRFSS (Table 3). Shenson [23] conducted reliability and validity testing of self-reported pneumococcal vaccination data from the BRFSS using test/retest methods. Seventy three percent of respondents provided identical information on vaccination when retested, two years after the initial data collection time period. Validity measures were conducted by comparing data provided by a small subset of BRFSS respondents with Medicare claims or medical records. Self-reports had a sensitivity of 0.75 and specificity of 0.83, within substantial levels of kappa. Bradbury [24] conducted test/retest comparisons of responses to questions regarding colorectal cancer testing for BRFSS respondents in Massachusetts. Overall, their research suggested that reliability was moderate to substantial (k = 0.55 to 0.69). Questions regarding having had tests were more reliable than questions regarding timing of such tests.

**Table 3:** Reliability and validity studies of immunization, preventive screening and testing estimates

**First author (year)**	**Focus of research**	**Research findings (confidence intervals)**
Shenson (2005)	Reliability and validity testing of immunization questions	Test/retest agreement on vaccination questions was 73%; Self-reports had a sensitivity of .75 and specificity of .83 when compared against medical records.
Bradbury (2005)	Test/ retest of colorectal cancer screening tests	Variation in reliability estimates due in part to time period between test/retest:
		***k*** = .55-.69
Cronin (2009)	Validity testing of mammography screening using registry rates and BRFSS rates	Estimates of BRFSS over reporting of mammography:
		16% women 40-49
		25% women 70-79
Fahimi (2008)	Validity testing of immunization questions from BRFSS, NHIS	Influenza vaccine prevalence:
		BRFSS (66.9-68.2)*
		NHIS (63.2-66.0)*
		Pneumonia vaccine prevalence:
		BRFSS (62.7-64.1)*
		NHIS (55.3-58.3)*

Cronin [25] tested validity of self-reports of mammography screening by comparing rates from BRFSS data with rates calculated by the NHIS and compared those rates to mammography registry data. They found that BRFSS estimates were similar to those reported by the NHIS. Both methods of self-reporting (BRFSS and NHIS) produced lower prevalence rates than registry rates. Fahimi [13] compared rates of vaccination for flu and pneumonia from the BRFSS and the NHIS. BRFSS respondents were more likely to report having had pneumonia and/or annual influenza vaccines than were respondents on the NHIS. Overall, reliability and validity studies on immunization, preventive screening and testing published since 2004 showed consistency across national surveys. Test/retest reliability indicated that similar answers were provided for some measures, even when two years elapsed between administrations of the survey. Respondents were better able to accurately recall that they had a preventative test than they were able to recall the dates of testing or screening.

### Physical activity measures

Questions on the BRFSS related to physical activity produced data that allow researchers to classify respondents into levels of recommended and vigorous physical activity, from inactive to vigorously active. Eight studies were identified from the literature which presented findings of reliability and/or validity of BRFSS physical activity measures (Table 4). Yore [26] conducted research including test/retest of the physical activity questions over a nine month period. Participants also were asked to maintain a log of physical activity and wore accelerometers to assess validity of self-reported responses. Log responses were more highly correlated with telephone self-reports than were measures taken from the accelerometer as the standard (k = 0.4 to 0.52 and 0.17 to 0.22, respectively). Reliability of data was also higher for those respondents who were in the vigorous activity category or when assessing responses related to strengthening. Validity was assessed by comparing telephone interview responses to log entries and accelerometer readings. Validity, using the log as a benchmark, ranged from k = 0.40 to 0.52, while validity estimates with the accelerometer as a base were lower at k = 0.17 to 0.22. The authors concluded that the validity and reliability of the BRFSS can be used to classify persons into groups of levels of activity. A second research effort by Yore and other colleagues [19] indicated that BRFSS occupational physical activity measures were highly replicated, especially when time between repeated measures was short. Overall, these publications supported the findings of the BRFSS at the moderate level of the kappa statistic. Substantial (k > 0.6) agreement was found when assessing reliability among persons who were categorized at the vigorous level of physical activity.

**Table 4:** Reliability and validity studies of physical activity estimates

**First author (year)**	**Focus of research**	**Research findings**
Yore (2007)	Reliability and validity using comparison with physical measures and repeated telephone interviews	Moderate activity group ***k*** = .35-.53
		Vigorous activity ***k*** = 80-.86;
		Recommended activity ***k*** = .67-.84;
		Strengthening measures ***k***= .85-.92
		Self-reports/ personal log ***k*** = .40 -.51
		Self-reports/accelerometer ***k*** = ≤ .31
Yore (2005)	Reliability using repeated measures and self-reported logs	1-5 days between surveys ***k*** = 1.0
		10-18 days between surveys ***k*** =.45
		10-19 days between surveys ***k*** =.40
Everson (2005)	Reliability using test/retest telephone surveys including gender and racial differences among indicators	Moderate activity ICC = .32-.58
		Vigorous activity ICC = .55-.85
		Leisure activity ICC = .46-.68
		Occupational activity ICC =.82
		Sedentary indicators ICC = .32-.90
Brown (2004)	Reliability using test/retest telephone surveys	Percent agreement for classification of active/insufficiently active/ sedentary 77.6;
		All activity groups ***k*** = .23-.56
		Walking measures ICC = .45
		Moderate activity ICC= .44
		Vigorous activity ICC= .39
Hutto (2008)	Reliability of questions when question order is changed	Question order effect BRFSS and alternate order, respectively
		Walking (37.7 , 41.0)
		Vigorous Activity (34.7, 37.0)
		Moderate Activity (40.3, 30.5)
		Meeting Physical Activity Recommendations (53.9, 51.7)
Pettee (2008)	Reliability of questions over different time periods	ICC= .42 and .55 for 3 week and 1 week test/retest on TV watching and physical activity question
Carlson (2009)	Validity testing comparing prevalence across surveys and methods	Active: mean difference BRFSS/ NHIS: 18.1
		Active: mean difference BRFSS/ NHANES: 14.8
		Inactive: mean difference BRFSS/ NHIS: 26.8
		Inactive: mean difference BRFSS/ NHANES: 18.5
Reis (2005)	Validity testing of multiple indicators from OPAQ to single question on the BRFSS	Agreement between single BRFSS occupational measure and OPAQ: ***k=*** .71

Everson and McGinn [10] reported reliability findings for test/retest physical activity responses by race and gender. They found some variability among race/gender groups. Their overall Inter-Correlation Coefficient (ICC) placing respondents into groups of vigorously active, moderately active or inactive activity ranged from 0.32 to 0.85. They also examined reliability for occupational and leisure measures of the BRFSS with ICCs ranging from 0.36 to 0.82. Sedentary indicators ranged from 0.32 to 0.83. Overall their study found fair to substantial reliability for measures tested. Brown [27] provided similar findings for test-retest methods, reporting “fair” to “substantial” ICC agreement, when measured using Landis and Koch’s [16] categories for kappa interpretation. Brown’s research also included percentage of respondents who were assigned to the same groups of levels of activity, based on responses to repeated BRFSS measures. Overall 77.6% of respondents were assigned to the same groups/levels of physical activity across repeated administrations of the survey. Hutto [28] found that overall, vigorous activity and walking were consistently reported even when question order was changed. However there were differences noted, especially for moderate physical activity when walking questions were posed prior to other activity questions. The authors recommended posing walking questions after moderate and vigorous physical activity questions in order to avoid bias in self-reporting. Pettee [18] found that a question from the BRFSS on television viewing held up well when a test/retest reliability study was conducted. ICCs for a one week retest were 0.55 and were at 0.42 for a three week retest schedule.

The BRFSS may also be compared with other surveys, interviews and physical measures taken of the same or similar populations. Carlson [29] conducted a review of prevalence estimates and trends of measures of physical activity across three surveys: the BRFSS, the NHIS and NHANES. As was noted earlier, the NHIS was conducted in face-to-face format, and NHANES combined face-to-face interviews supplemented with physical measures. The surveys also differed in the number and detail of physical activities responses collected. Levels of reported physical activity were higher for the BRFSS, than for the other two surveys. For example, the percentage of persons estimated to be “active” was 30.2 for the NHIS, 33.5 for the NHANES and 48.3 for the BRFSS. The three surveys were in agreement when trends were assessed, with higher levels of activity being associated with younger and among white, non-Hispanic respondents. These differences may be caused by the fact that the BRFSS included more measures of physical activity than the other surveys.

Reis [30] tested a self-assessment of physical activity related to work, the occupational physical activity questionnaire (OPAQ), which they correlated with the single occupational question from the BRFSS. Research participants provided information through self-reports and physical measures. Information was also collected through accelerometers worn by study participants. Reis found substantial agreement (k = 0.71) between the aggregated measures of occupational physical activity on the OPAQ and the BRFSS.

Overall, the identified studies of reliability and validity for physical activity measures supported findings of the BRFSS. The reliability of indicators was supported using test/retest methods and time trend methods. Reliability measures for physical activity questions were found to be in the fair to substantial ranges of the statistic k. Findings indicate that the most reliable estimates were achieved for persons who exercise regularly. Validity was assessed by comparison with other surveys, although some of the comparison surveys used different data collection methods. Some research also compared BRFSS physical activity measures and responses to physical measures such as accelerometers. Variation of prevalence estimates was found in some instances, but trends were similar when comparing among survey results over time.

It is not surprising to find that differences in reporting physical activity change over time. Respondents who were contacted for test/retest studies may have, in fact, changed their levels of activity in the interim between testing. Therefore, higher levels of reliability of measures in shorter term retests are reasonable.

### Chronic conditions and mental health measures

The BRFSS collected data on a number of chronic conditions, including diabetes, asthma, arthritis, and cardiovascular diseases. Fahimi [13] compared prevalence levels of diabetes and asthma among BRFSS, NHIS and NHANES. NHIS and BRFSS estimates were similar, with NHANES estimates showing significant differences (Table 5). When asked whether they had been told that they have diabetes, respondents to the BRFSS and NHIS had similar prevalence estimates (confidence intervals of 7.9 to 8.1 and 7.8 to 8.5, respectively). NHANES estimates on this question ranged from 5.1 to 7.4. A similar question on asthma diagnosis resulted in more variance between BRFSS and NHIS (confidence intervals of BRFSS 13.1 to13.6; NHIS 9.5 to 10.3). 

**Table 5:** Reliability and validity studies of chronic condition estimates

**First author (year)**	**Focus of research**	**Research findings (confidence intervals)**
Fahimi (2008)	Comparison of BRFSS, NHIS and NHANES prevalence estimates	Diabetes
		BRFSS (7.9-8.1)*
		NHIS (7.8-8.5)*
		NHANES(5.1-7.4)*
		Asthma
		BRFSS (13.1-13.6)*
		NHIS (9.5-10.3)*
Bombard (2005)	Validity and reliability of arthritis questions using different modes and physical measures	Sensitivity 70.8%
		Specificity 70.3%
		Agreement between phone and written responses ***k*** = .68
Sacks (2005)	Validity of BRFSS arthritis questions using physical measures	For ages 45-64
		Sensitivity 77.4%
		Specificity 58.8%
		For ages 65 and older
		Sensitivity 83.6%
		Specificity 70.6%
Cossman (2008)	Validity of BRFSS cardiovascular measures using prescription data	Correlation coefficients (r) = .43-.66

Bombard [31] conducted a validity and reliability study of BRFSS arthritis questions among seniors. Telephone responses were compared to written medical history and physical examination information for a select group of study participants. Agreement between the modes of self-reports was high (k = 0.68) and sensitivity and specificity of the questions as compared to the physical measures was 70.8 and 70.3%, respectively. Sacks [32] also conducted validity tests of arthritis questions using physical measures. Persons who had upcoming appointments were asked BRFSS questions prior to physical examinations. Self-assessments were found to be more accurate among older respondents. Sensitivity and specificity were at 77.4 and 58.8%, respectively for persons aged 45 to 64 and sensitivity and specificity at 83.6 and 70.6% for participants over 64 years of age. Cossman [33] used data on prescription drugs for treatment of cardiovascular disease as a proxy for prevalence. They then compared prevalence rates using these data on a substate/ county level to prevalence rates produced by the BRFSS. Correlation coefficients ranged from 0.43 to 0.66 (moderate to strong) for the area within twenty-four states where BRFSS modules on cardiovascular disease were administered.

The BRFSS included a number of quality of life and related mental health. Andresen [34] conducted test/retest responses questions among Missouri respondents (Table 6). They found moderate to excellent reliability across quality of life measures, with only slight variation in categorical (when compared to continuous variables) measures and among older respondents. Self-reported overall health measures reliability was substantial (k = 0.75) as were measures of poor physical health days (k = 0.71), poor mental health days (k = 0.67), limited activity days (k = 0.57), healthy days (k = 0.75), frequent mental distress (k = 0.58) and frequent physical distress (k = 0.64). Variation was also greater when time between test/retest measures was longer. Kapp [35] conducted a similar study within the same state (Missouri). The authors compared item reliability for all respondents and for cancer survivors. Kappa statistics for all measures and groups tested were within the moderate to substantial range (k = 0.43 to 0.80) and found the measures to be appropriate quality of life indicators among cancer survivors.

**Table 6:** Reliability and validity studies of mental health estimates

**First author (year)**	**Focus of research**	**Research findings**
Andresen (2003)	Reliability test/retest of quality of life measures among Missouri respondents	Overall health (***k***= .75)
		Poor physical health days (***k***= .71)
		Poor mental health days (***k***= .67)
		Limited activity days (***k***= .57)
		Healthy days (***k***= .75)
		Frequent mental distress (***k***= .58)
		Frequent physical distress (***k***= .64)
Kapp (2009)	Test/retest of quality of life measures among cancer survivors and other respondents	Physical distress(***k***=.72)
		Activity limitation (***k***=.75)
		Social and emotional support (***k***=.57)
		Life satisfaction (***k***=.61)
		Pain (***k***=.75)
		General health (***k***=.65)

### Behavioral health risks/status

Three components of behavioral health and status (overweight and obesity, tobacco use and alcohol use) are examined in this section. A comprehensive study of multiple indicators from BRFSS, NHANES and NHIS was conducted by Fahimi [13]. These authors found that the BRFSS prevalence measures of obesity were statistically similar to those of NHIS (Table 7). Observed differences between BRFSS and self-reports from the NHANES were small. As with the two previously cited studies, height was less likely to be biased than was weight.

**Table 7:** Reliability and validity studies of obesity estimates

**First author (year)**	**Focus of research**	**Research findings**
Fahimi (2008)	Comparison of BRFSS, NHANES, and NHIS measures of height and weight	NHIS and BRFSS height measures differed by .14 inches
		NHIS and BRFSS weight measures differed by 1.2%
		BRFSS and NHANES height measures were statistically identical
		BRFSS weight measures fell between measures taken by NHANES (self-reports) and NHIS
Ezzati (2006)	Weighting BRFSS self-reports of height and weight by NHANES to correct for bias/ underestimation	BRFSS underestimation from 1999–2002 averaged 5.9%, but could be corrected by weighting
Yun (2006)	Weighting BRFSS self-reports of height and weight by NHANES to correct for bias/ underestimation by race, gender and age	BRFSS underestimated prevalence of obesity and overweight groups by 9.5 and 5.7 percentage points, respectively, Estimates for femalesaged 20–39 differed from NHANES physical measures most often.

Prevalence estimates reported by Ezzati [36] found that while bias in self-reported height and weight estimates were found, especially among women, these biases could be corrected through the use of weighting. These scholars used NHANES to determine benchmarks for regions and states then adjusted BRFSS data accordingly. They concluded that telephone survey respondents provided data that underreported Body Mass Index (BMI) but that BMI data were useful when appropriately weighted. Since telephone survey data are less expensive to collect, the authors found this method to be acceptable to ascertain national and sub-national prevalence estimates of obesity. Yun [37] found similar results, noting that self-reported biases were not consistent across demographic groups, and that appropriate weighting is necessary to correct for demographic factors such as gender and educational attainment. Their findings indicated that prevalence of obesity and overweight was underreported by between 9.5 and 5.7 percentage points. Underestimation was particularly noted among 20–39 year old females.

Although tobacco use is widely noted to be related to health status, there are relatively few comparative studies published since 2004 concerning reliability of tobacco use prevalence measures across national surveys. This may be due to the fact that question format differs on these studies, making them somewhat difficult to compare. The BRFSS, NHIS and NHANES all measured tobacco use in some way. Klein [38] included a fourth survey, the Harris poll online (HPOL) a non-random web-based sample survey of over 100,000 respondents, to review differences among national survey estimates of tobacco use prevalence (Table 8). After weighting, they found that BRFSS (using national median of the state BRFSS surveys) and NHIS estimates were statistically similar, and that NHANES estimates were slightly higher. The HPOL results, taken from nonrandom samples, differed slightly from NHANES, BRFSS and NHIS findings. The authors concluded that self-reports varied by methodology and question format, but that measures from all surveys produced utile information for researchers.

**Table 8:** Reliability and validity studies of tobacco and alcohol use estimates

**First author (year)**	**Focus of research**	**Research findings (confidence intervals)**
Klein (2007)	Validity comparison of online, personal interview, examination and telephone survey results of tobacco use	Smoking prevalence
		BRFSS 20.9 (median)
		NHIS 20.9-22.1
		NHANES self-reports 22.4-27.5
		NHANES physical measures 30.6-38.1
		HPOL 23.7-24.4
Fahimi (2008)	Validity test comparing three national surveys	Current smoker prevalence estimates:
		BRFSS (20.4-21.0)*
		NHIS (20.3-21.6)*
		NHANES (21.4-25.9)*
Fahimi (2008)	Validity test comparing national surveys	Binge drinking prevalence estimates:
		BRFSS (4.2-4.4)*
		NHIS (4.5-4.9)*
		Average Number of drinks per occasion:
		BRFSS (2.4-2.5)*
		NHIS (2.4-2.5)*
Miller (2004)	Comparison of in-home and telephone survey results related to adult binge drinking	Binge drinking state level prevalence estimates:
		NSDUH (21.2-22.0)*
		BRFSS (14.5-15.5)*
		Absolute differences by race, age, gender groups for national prevalence estimate:
		(.06-8.1)*

Fahimi [13] compared national survey data from 2006 for NHANES, NHIS and BRFSS. They calculated confidence intervals for current smoking and found no statistical differences between confidence intervals of prevalence estimates from BRFSS (20.4 to 21.0) and NHIS (20.3 to 21.6), but higher levels of prevalence reported by NHANES (21.4 to 25.9). Fahimi’s research included a number of comparisons of BRFSS and NHIS prevalence estimates related to alcohol consumption. These questions were not asked of NHANES participants. NHIS and BRFSS surveys included questions on drinking which differed in format. The NHIS question provided information on self-reported consumption of 5 or more drinks in one day, while the BRFSS self-reports use total number of drinks in a single occasion to determine binge drinking. Although NHIS question did not measure binge drinking, estimates were statistically similar for binge drinking and average number of drinks, despite question wording differences. BRFSS respondents were classified into groups of persons who drink five or more drinks on “one occasion,” while NHIS respondents were grouped into categories which include five or more drinks “in one day”. Miller [39] compared state-level prevalence estimates from the BRFSS and NSDUH. The NSDUH differed in method from the BRFSS in that it was conducted as a face-to-face interview in respondents’ homes. These researchers combined data from 1999 and 2001 from the BRFSS. Eight states with large enough samples of NSDUH data were used to review state-level prevalence rates. Despite some variance in prevalence for individual states and some demographic categories, characteristics of binge drinkers between the two surveys were similar.

### Health risk, injury risk and sexual behavior measures

Only two studies published since 2004 were identified which examined reliability and/or validity of BRFSS measures of health risks related to sexual behavior (Table 9). Santelli [40] compared estimates taken from female respondents to the NSFG and the BRFSS related to contraception and reasons for nonuse. Since question format differed on these two surveys, recoding of some variables was conducted to make measures more consistent. The overall percentage of women who were not sexually active was higher for the BRFSS (16%) when compared to the NSFG (12.5%). Many measures of contraception were the same on the two surveys, but small, statistically significant differences were found for vasectomy (7.7 and 6.3%), use of the pill (21.9 and 19.6%), rhythm (1.5 and 1.0%), use of a diaphragm (0.5 and 0.2%), and withdrawal (0.3 and 2.7%) for the BRFSS and the NSGF, respectively. Fahimi [13] found significant differences between BRFSS and NHIS respondents when data from HIV testing was reviewed. BRFSS respondents were more likely (confidence interval of 43.4 to 44.2) to report having had an HIV test than were NHIS respondents (confidence interval of 33.9 to 35.3).

**Table 9:** Reliability and validity studies of health risks related to sexual behavior, injury rick and partner violence

**First author (year)**	**Focus of research**	**Research findings (confidence intervals)**
Santelli (2008)	Validity of BRFSS using comparison with NSFG	BRFSS and NSGF, respectively
		Not Sexually Active (16.5% and 12.5%)
		Vasectomy (7.7% and 6.3%)
		Use of the pill (21.9% and 19.6%)
		Rhythm (1.5% and 1.0%)
		Diaphragm (.5% and .2%)
		Withdrawal (.3% and 2.7%)
Fahimi (2008)	Comparison of BRFSS and NHIS prevalence estimates	BRFSS (43.4-44.2)*
		NHIS (33.9-35.3)*
Bonomi (2006)	Validity testing of BRFSS and WEB surveys	Agreement levels BRFSS/ WEB
		Any abuse (88.2%)
		Sexual abuse (93.6%)
		Physical abuse (90.7%)
		Fear due to threats (92.9%)
		Controlling behavior (91.9%)

Only one study published since 2004 was identified which examined reliability of BRFSS measures on violence and injury risk (Table 9). Bonomi [41]) used questions from the BRFSS and the women’s experience with battering scale (WEB) to determine the relationship between the sets of questions from the two surveys. Information was taken from a separate administration of BRFSS questions to a sample of women enrolled in a health cooperative. Data from the regularly implemented BRFSS were not used. The authors then noted when each of the surveys classified the women from the health cooperative as abused or not abused. Agreement levels between the two sets of surveys questions were high for any abuse (88.2%), sexual abuse (93.6%), physical abuse (90.7%), fear due to threats (92.9%) and controlling behavior (91.9%). Overall the BRFSS reported a higher level of abuse than did the WEB.

## Discussion

Despite concerns about declines in telephone survey response rates, the BRFSS is comparable to other national and state level surveys investigating similar topics. In comparison with the last comprehensive review of literature on reliability and validity conducted over a decade ago [4], few data quality differences were noted. While the BRFSS was found to be reliable and to have high overall levels of validity when compared to other surveys in this review, differences were more often noted for validity than for reliability. There are many reasons why responses may differ over time or prevalence rates differ among large surveys. Comparison of BRFSS data with that of other surveys is likely to show the effects of differences in the wording of questions, the number of questions focusing on a single topic or measure, survey mode and/ or the length of the questionnaire. Moreover, questions of a sensitive nature (for example, questions related to binge drinking and/or HIV testing) differed in the mode of their administration [41]. In many cases questions on these large scale surveys differed in format and/or in categories for closed-ended questions. For example, the questions on physical activity from the BRFSS and the NHIS differ in both number and format. Therefore, prevalence estimates should be expected to differ due to question wording as well as mode. Sampling is also a likely cause of prevalence differences. All surveys require that subjects agree to be part of the sample. Recruitment of persons to take part in telephone surveys, in-person interviews, web-based surveys, written surveys and physical measures examinations are all presented here. The burden on respondents is greater for face-to-face interviews and greatest for physical examination. There are likely to be differences in health indicators among recruited subjects in each of these modes of data collection. Other surveys aggregate relatively smaller samples from a number of areas and weight responses using demographic characteristics to produce national prevalence estimates. Therefore it is not surprising that prevalence rates varied somewhat from one survey to another.

In some cases, even when prevalence estimates differed, other statistical relationships within survey datasets remained the same. For example, although rates of binge drinking were different among some of the surveys, demographic characteristics associated with binge drinking persisted for all of the datasets examined by the literature cited here. In other cases where prevalence rates differed, trends noted in the BRFSS were also noted in other national surveys. Over or under reporting of health risk behaviors is in part a function of the desire of respondents to please interviewers, regardless of whether responses were collected by phone or in personal interviews. However, bias created by the physical presence of interviewers is likely to be stronger than that created by surveys conducted over the phone when respondents were asked sensitive questions [41]. In other cases differences in prevalence may have been due to actual changes in health risk behaviors during the intervening period between test and retest in reliability studies, and therefore not indicative of measurement error. As was noted in the studies of reliability of physical activity measures, respondents may have actually changed their levels of physical activity in the intervening period. Differences between self-reports of chronic conditions and physical measures may be a function of respondents who are not aware of their presence, which become evident when physical measures are taken. For example, self-reports rely on diagnoses of chronic conditions such as diabetes or hypertension. Respondents may accurately report whether they have ever been diagnosed with these conditions, while at the same time be unaware of their current presence. This was supported by data showing BRFSS estimates to be reliable, but to differ from physical measure surveys.

## Conclusions

The BRFSS produced similar prevalence rates as other surveys examined by the literature; however, care should always be taken when comparing estimates from different surveys. Consumers of information should examine the questionnaires, the number and timing of questions as well as the mode of interview and sampling methods before determining that prevalence rates are comparable. As BRFSS has moved to a new weighting method and included cell phone respondents in its sample, users should replicate their examination of reliability and validity of BRFSS estimates. This research updated that of Nelson [4] completed more than a decade ago, but results are similar in that research on BRFSS reliability and validity continues to support the utility of the data. This review of literature also indicates that there are many opportunities for continued research in this area, especially with the release of cell phone data and new weighting methods at BRFSS in recent months. The paucity of data quality information in some health topic areas calls for additional research on the reliability of indicators and estimates across surveys.

## Abbreviations

BMI: Body Mass Index; BRFSS: Behavioral Risk Factor Surveillance System; CDC: Centers for Disease Control and Prevention; CPS: Current Population Survey; HMO: Health Maintenance Organization; HPOL: Harris Poll Online; ICC: Intercorrelation coefficient; NHANES: National Health and Nutrition Examination Survey; NHIS: National Health Interview Survey; NSFG: National Survey of Family Growth; NSDUH: National Survey of Drug Use and Health; OPAQ: Occupational Physical Activity Questionnaire; WEB: Women’s Experience with Battering Scale.

## Competing interests

The authors know of no competing interests.

## Authors’ contributions

Carol Pierannunzi participated in the literature review, completed the first draft; Sean Hu participated in the literature review and commented on drafts of the manuscript; Lina Balluz had the original concept of the manuscript and commented in drafts. All authors participated in responding to reviewers’ comments and suggestions for change.

## Pre-publication history

The pre-publication history for this paper can be accessed here:

http://www.biomedcentral.com/1471-2288/13/49/prepub
